# Transcriptomic characterization of the molecular mechanisms induced by RGMa during skeletal muscle nuclei accretion and hypertrophy

**DOI:** 10.1186/s12864-022-08396-w

**Published:** 2022-03-07

**Authors:** Aline Gonçalves Lio Copola, Íria Gabriela Dias dos Santos, Luiz Lehmann Coutinho, Luiz Eduardo Vieira Del-Bem, Paulo Henrique de Almeida Campos-Junior, Izabela Mamede Costa Andrade da Conceição, Júlia Meireles Nogueira, Alinne do Carmo Costa, Gerluza Aparecida Borges Silva, Erika Cristina Jorge

**Affiliations:** 1 Departamento de Morfologia, Instituto de Ciências Biológicas, Universidade Federal de Minas Gerais, Av Antonio Carlos, 6627, Pampulha, Belo Horizonte, Minas Gerais 31.270-901 Brasil; 2 Departamento de Zootecnia, Escola Superior de Agricultura Luiz de Queiroz, Universidade de São Paulo, Piracicaba, Brasil; 3 Departamento de Botânica, Instituto de Ciências Biológicas, Universidade Federal de Minas Gerais, Belo Horizonte, Brasil; 4 Departamento de Ciências Naturais, Universidade Federal de São João del Rei, São João del Rei, Brasil; 5 Departamento de Bioquímica E Imunologia, Instituto de Ciências Biológicas, Universidade Federal de Minas Gerais, Belo Horizonte, Brasil

**Keywords:** Axon Guidance, Myogenesis, Hypertrophy, Hyperplasia, Skeletal muscle differentiation, Transcriptomic analysis

## Abstract

**Background:**

The repulsive guidance molecule a (RGMa) is a GPI-anchor axon guidance molecule first found to play important roles during neuronal development. RGMa expression patterns and signaling pathways via Neogenin and/or as BMP coreceptors indicated that this axon guidance molecule could also be working in other processes and diseases, including during myogenesis. Previous works from our research group have consistently shown that RGMa is expressed in skeletal muscle cells and that its overexpression induces both nuclei accretion and hypertrophy in muscle cell lineages. However, the cellular components and molecular mechanisms induced by RGMa during the differentiation of skeletal muscle cells are poorly understood. In this work, the global transcription expression profile of RGMa-treated C2C12 myoblasts during the differentiation stage, obtained by RNA-seq, were reported.

**Results:**

RGMa treatment could modulate the expression pattern of 2,195 transcripts in C2C12 skeletal muscle, with 943 upregulated and 1,252 downregulated. Among them, RGMa interfered with the expression of several RNA types, including categories related to the regulation of RNA splicing and degradation. The data also suggested that nuclei accretion induced by RGMa could be due to their capacity to induce the expression of transcripts related to ‘adherens junsctions’ and ‘extracellular-cell adhesion’, while RGMa effects on muscle hypertrophy might be due to (i) the activation of the mTOR-Akt independent axis and (ii) the regulation of the expression of transcripts related to atrophy. Finally, RGMa induced the expression of transcripts that encode skeletal muscle structural proteins, especially from sarcolemma and also those associated with striated muscle cell differentiation.

**Conclusions:**

These results provide comprehensive knowledge of skeletal muscle transcript changes and pathways in response to RGMa.

**Supplementary Information:**

The online version contains supplementary material available at 10.1186/s12864-022-08396-w.

## Background

Repulsive guidance molecule a (RGMa) comprises the first repulsive glycoprotein member identified in the family of repulsive guidance molecules [1]. It was originally identified as a repulsive clue in the orientation of axonal growth in the central and peripheral nervous system and as an important target for neuronal survival [1–4] However, RGMa action domains were found to go beyond the processes related to neurogenesis and could be extended to different processes, including the induction of endochondral ossification during skeletal development [5], the suppression of endothelial tube formation [6], and inflammatory responses [7, 8].

These diverse functions can be performed by RGMa because it can signal through different receptors and work as a modular protein. The RGMa C-terminal domain (C-RGMa) harbours a GPI-anchor and presents affinity to the type I transmembrane neogenin receptor [9, 10], which is known as a guidance receptor for migrating neuronal and mesodermal cells [11–13]. This domain also harbours a von Willibrand type D structural domain, containing a GDPH autocatalytic site [14]. The RGMa N-terminal domain (N-RGMa) harbours a signal peptide, an additional neogenin-binding site, and an RGD motif, that is known to be important in cell–cell adhesion processes mediated by integrins [15]. However, RGMa signaling through integrins has not been reported thus far. Notably, N-RGMa presents high affinity to bone morphogenetic proteins (BMP) ligands, making RGMa (and all the members of this family) a modulator of this important signaling pathway [16–19]. N-RGMa shares the same binding site on the BMP ligand with the ectodomain of the BMP type I receptor A (BMP-R1A), meaning that RGM can induce the BMP canonical signaling pathway via activation of Smad 1/5/8. RGMa could also integrate neogenin and BMP signaling cascades [5, 20–22]. Finally, RGMa was recently found to promote astrogliosis and glial scar formation in a rat model of middle cerebral artery occlusion/reperfusion by forming a complex with ALK5 and Smad2/3, which are the main members of the transforming growth factor β1 (TGFβ1) signaling pathway [23].

In previous works, we found *RGMa* transcripts in the myogenic and satellite cell precursors in the somites during chicken embryonic development [24] and at the sarcolemma and in the sarcoplasm of adult mice muscle cells [25]. RGMa overexpression in C2C12 cells induced the formation of larger myotubes (hypertrophy) with an increased number of myonuclei (nuclei accretion), while its knockdown resulted in the appearance of smaller cells, with a deficient ability to form multinucleated myotubes [25].

Skeletal muscle cell size is known to be determined by the balance between protein and cellular turnover [26–28]. Because of cellular turnover, the skeletal muscle cell grows by myonuclei accretion, in a process mediated by cell fusion. The increase of myonuclei into myofibers leads to muscle mass expansion due to the higher rate of transcription given the nuclear turnover [29]. Muscle nuclei accretion is important not only during embryonic development but also during muscle regeneration [29–36]. In contrast, because of protein turnover, the skeletal muscle cell grows by upregulating protein synthesis pathways, consequently increasing the level of protein within the muscle tissue [27, 29]. Although hypertrophy and nuclei accretion are two distinct processes, they frequently occur together [36], and not all signals involved during the proliferation and differentiation of skeletal musculature are known.

Despite having found that RGMa can induce hypertrophy and nuclear accretion in skeletal muscle cells cultivated in vitro, the molecular mechanisms that are induced by this axon guidance molecule in these particular cells have not been investigated thus far. Our hypothesis is that RGMa can modulate the expression of a number of transcripts in skeletal muscle cells, especially those involved with nuclei accretion and striated muscle cell differentiation. In this work, C2C12 cells were treated with RGMa recombinant protein to investigate the molecular mechanisms that are modulated by this axon guidance molecule during myogenic differentiation. This was the first work to show, through RNA-seq analysis, the transcript targets and molecular profile triggered by RGMa during skeletal muscle differentiation and its possible involvement in multiple functions, including cell fusion and hypertrophy.

## Results

### Overview of the RNA-seq data and differentially expressed transcripts (DETs)

**Fig. 1:**
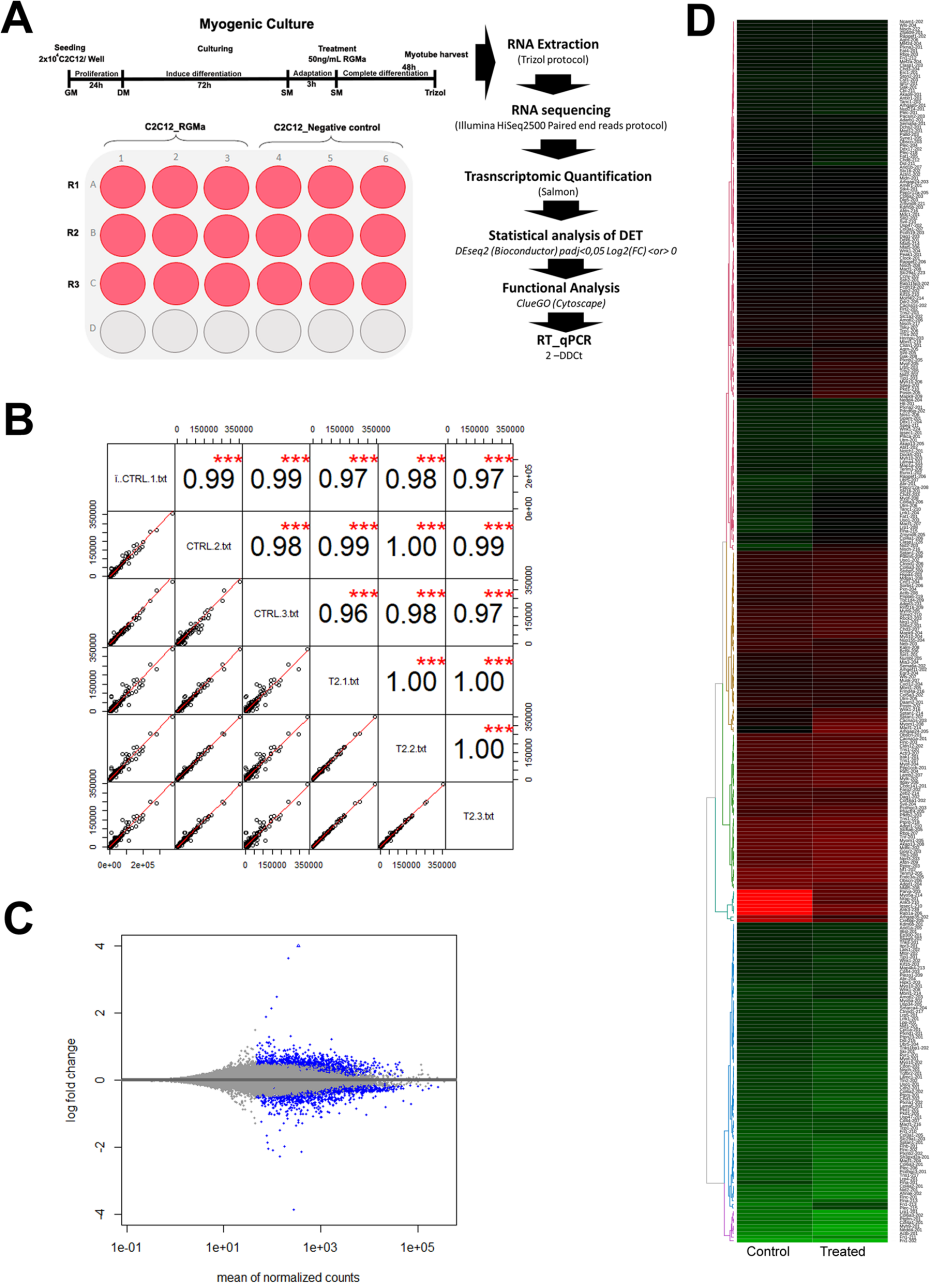
Quality and transcriptomic profile of RGMa-treated myoblasts during myogenic differentiation. **A** Experimental design. **B** Pearson Correlation Coefficient (PCC) analysis of normalized read-counts denoted a high internal consistency and reproducibility of treated and control replicates. **C** MA plot analysis showing the RNA-seq profile of the log_2_ (fold change) distributions of all DETs in the average of normalised counts. Each point represents one transcript. Those dots marked in blue were detected as differentially expressed at a 5% FDR with log_2_(FC) > 0 (upregulated) and log_2_(FC) < 0 (downregulated) after RGMa treatment. Transcripts with similar expression levels are represented around the horizontal line (y = 0). Dots that are outside the window are plotted as triangles. **D** Heatmap analysis of DET with muscle-associated terms (‘cellular component,’ ‘biological process,’ and ‘molecular function’) of Gene Ontology (GO). Transcripts with the lowest expression values are marked in red, median expression values in black, and the highest expression values in green

The quality of the generated sequence database was first evaluated to verify the internal consistency and reproducibility of the replicate samples, as well as the disparity among them. The Pearson correlation coefficient (PCC) of the normalized read-counts revealed a perfect positive linear correlation between all RGMa-treated samples and an extremely strong correlation among the control ones (Fig. 1A). The analysis also revealed a subtle difference between treated and control samples, as there was a positive linear correlation showing Pearson *r* coefficients above 0.97 among all correlated samples (Fig. 1B). MA-plot analysis revealed that RGMa treatment modulated gene expression in skeletal muscle cells, with very few of them presenting a drastic effect (Fig. 1C).

The expression of 23,855 transcripts could be detected after normalization, and 2,195 were found as differentially expressed transcripts (DETs, *p* < 0.05, Fig. 1B, grey dots), with 943 upregulated and 1,252 downregulated by RGMa treatment compared to the control (Fig. 1C, blue dots with Log2(FC) > 0 and Log2(FC) < 0, respectively). Twenty-six DETs were exclusively expressed in RGMa-treated myoblasts, and 79 DETs had their expression drastically altered by the treatment (Supplementary Table 1). Differential expression analysis was also performed in gene level. We found that RGMa could modulate the expression of 1,788 genes (DEGs, *p* < 0.05, Supplementary Table 2). From the 2,195 DETs, 1,091 were also found as DEGs, meaning that 1,104 (~ 50%) were found as differentially expressed only at the transcription level.

The most drastic effects among the DETs were also observed as a heatmap of transcripts with enriched muscle-associated terms (Fig. 1D). The heatmap also allowed the observation that the expression of the majority of the transcripts did not change considerably between the control and treated samples. The 20 most upregulated and 20 most downregulated transcripts by RGMa treatment in C2C12 cells are shown in Table 1.

**Table 1:** The most drastically altered transcripts in RGMa-treated C2C12 myoblast, during myogenic differentiation

Transcripts most downregulated by RGMa			Transcripts most upregulated by RGMa		
**Ensembl Transcript Access**	**Trancript name**	**log2(FC) < 0**	**Ensembl Transcript Access**	**Trancript name**	**log2(FC) > 0**
ENSMUST00000113926.7	Zfx-203	-12,09,700,663	ENSMUST00000160260.8	Pou2f1-208	11,86,541,962
ENSMUST00000187142.1	Zfp469-202	-11,6,517,089	ENSMUST00000075836.11	Dock7-202	11,30,650,103
ENSMUST00000111427.8	Pou2f1-205	-11,58,532,487	ENSMUST00000182593.7	Prrc2c-209	11,18,773,896
ENSMUST00000113870.2	Tsc1-204	-11,08,073,619	ENSMUST00000182155.7	Ank3-210	10,39,693,989
ENSMUST00000169353.2	Kifc3-202	-10,96,464,702	ENSMUST00000040711.14	Nrap-201	10,01,424,135
ENSMUST00000177916.7	Zfp131-201	-10,66,135,536	ENSMUST00000106643.7	Parva-203	9,856,496,026
ENSMUST00000134230.7	Hnrnph1-211	-10,61,576,893	ENSMUST00000155282.8	Myo5a-214	9,842,528,635
ENSMUST00000107857.10	Ap2a1-202	-10,25,950,022	ENSMUST00000097864.8	Pum1-203	9,697,390,548
ENSMUST00000194801.5	Rbm5-224	-10,17,739,189	ENSMUST00000212451.1	Mau2-206	9,650,005,064
ENSMUST00000132947.1	Pds5b-204	-9,920,365,984	ENSMUST00000217647.1	Scaper-205	9,631,678,893
ENSMUST00000154403.7	Polg-214	-9,869,641,974	ENSMUST00000212100.1	Iqsec1-210	9,270,515,836
ENSMUST00000170647.1	Tnpo3-209	-9,791,146,902	ENSMUST00000039892.8	Tbc1d25-201	9,251,526,431
ENSMUST00000231973.1	D16Ertd472e-205	-9,770,163,529	ENSMUST00000183148.7	Ank3-239	9,209,545,896
ENSMUST00000095037.1	Whrn-204	-9,756,960,752	ENSMUST00000163483.1	Rab1a-206	9,145,085,159
ENSMUST00000208730.1	Picalm-212	-9,589,780,073	ENSMUST00000230614.1	Acap2-203	9,047,789,318
ENSMUST00000066986.12	Zfp142-202	-9,460,284,761	ENSMUST00000171937.1	Arhgap35-202	6,619,772,687
ENSMUST00000222395.1	Atg2b-205	-9,411,773,318	ENSMUST00000205765.1	Crebbp-205	6,563,438,613
ENSMUST00000150905.1	Htra1-204	-9,363,035,965	ENSMUST00000224799.1	Spire1-207	5,741,134,073
ENSMUST00000092614.8	Pcgf1-201	-9,362,877,176	ENSMUST00000098816.9	Slc7a2-202	4,478,565,603
ENSMUST00000216284.1	Cep164-207	-4,150,389,783	ENSMUST00000194877.5	Ints7-206	4,424,334,603

The twenty most highly downregulated (Log2(Fold Change) < 0) and twenty most highly upregulated (Log2(Fold Change) > 0) Differentially Expression Transcripts (DET—with a false discovery rate (FDR) < 0,05) modulated in C2C12 cells treated with RGMa during differentiation. Access number and transcript name identified in the Ensembl database; log2(FD) < 0 corresponds to the fold change of the downregulation and log2(FD) > 0, of the upregulation of each transcript after RGMa treatment

The most highly upregulated DET induced by RGMa treatment was the *Pou2F1* transcription factor (isoform Pou2F1-208, ENSMUST00000160260.9), also known as Oct-1 (Table 1). Among the other highly expressed genes, RGMa was able to induce the expression of genes related to skeletal muscle structure, including sarcomere and costamere organization (e.g., *Ank3*, *Nrap* and *Parva*), vesicle formation and trafficking (e.g., *Myo5a*, *Iqsec1*, *Tbc1d25*, *Acap* and *Rab1a*), and control of the cell cycle (*Mau2* and *Scaper*).

Notably, another isoform of the *Pou2F1* transcription factor (Pou2F1-205, ENSMUST00000111427.9) was found to be one of the most downregulated genes by RGMa treatment (Table 1).

Among the others, the downregulation of genes from the same categories included those associated with regulators of muscle mass and structure, such as *Tsc1* and *Tnpo3*, with the formation of clathrin-coated vesicles (*Ap2a1,* and *Picalm*), and with cell cycle progression and apoptosis (*Pcgf1, Kifc3 and Cep164*) (Table 1).

### RNA categories among DET

**Fig. 2:**
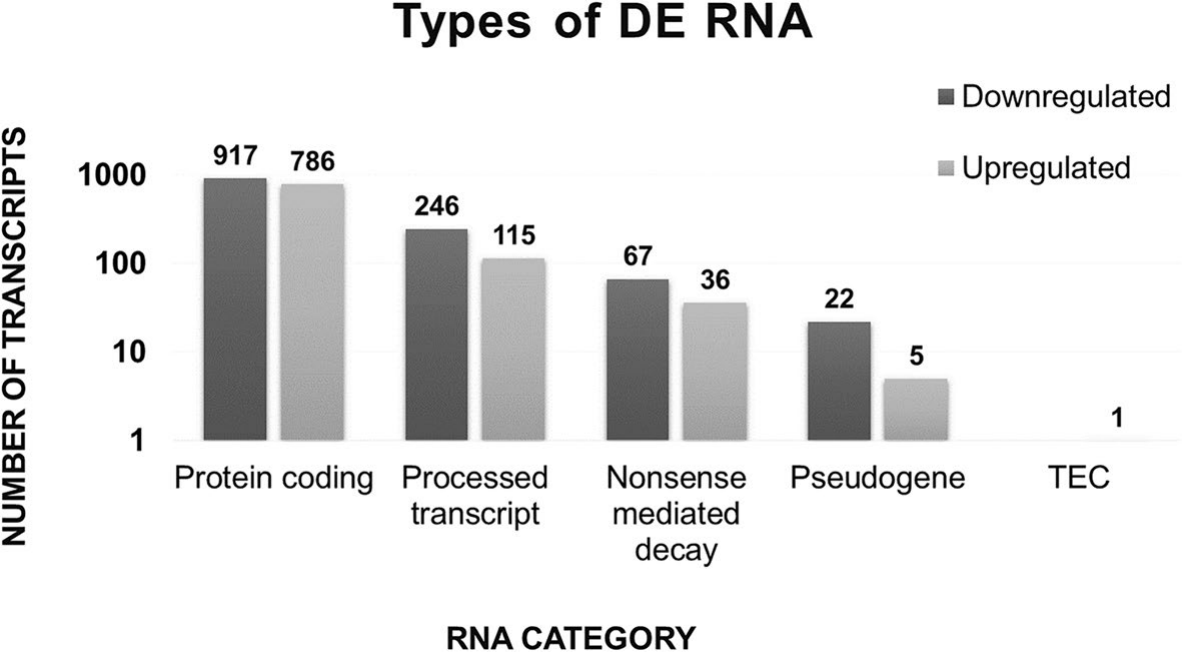
RNA biotypes modulated by RGMa treatment. RGMa could modulate the differential expression of 13 RNA biotypes, classified in six RNA categories according to Ensembl (https://m.ensembl.org/info/genome/genebuild/biotypes.html): (1) protein coding, (2) processed transcripts (*lncRNA: antisense, bidirection-promoter-lncRNA, lincRNA, retained intron* and *ncRNA: snRNA and Mt-rRNA*), (3) nonsense mediated decay, (4) pseudogenes (*processed-pseudogenes, transcribed-processed-pseudogene, and unprocessed-pseudogene*), and (5) Tec (to be experimentally confirmed)

Given the reliability of the transcriptome data, we next classified all 2,195 DETs by RNA biotypes to determine which were the main RNA categories influenced by RGMa treatment. Among the 1,252 DETs that were found to be downregulated, 917 (73.2%) were protein coding, 246 (19.6%) were processed transcripts, 67 (5.35%) were NMDs, and 22 (1.75%) were pseudogenes (Fig. 2). Among the 943 upregulated DETs, 786 (83.3%) were protein coding, 115 (12.2%) were processed transcripts, 36 (3.8%) were NMDs, 5 (0.5%) were pseudogenes, and 1 (0.1%) was a TEC (Fig. 2). Overall, this data revealed that most of the RNA biotypes that were modulated by RGMa treatment were ORF-containing RNAs, while the remaining were composed of RNAs mainly associated with the regulation of gene expression, including the NMD category, which was composed of transcripts containing a premature stop codon, and processed transcripts, a category composed of lncRNA, ncRNA, antisense, and intron-retained RNAs.

### GO pathway enrichment analysis of the non-coding RNA found as DETs

**Fig. 3:**
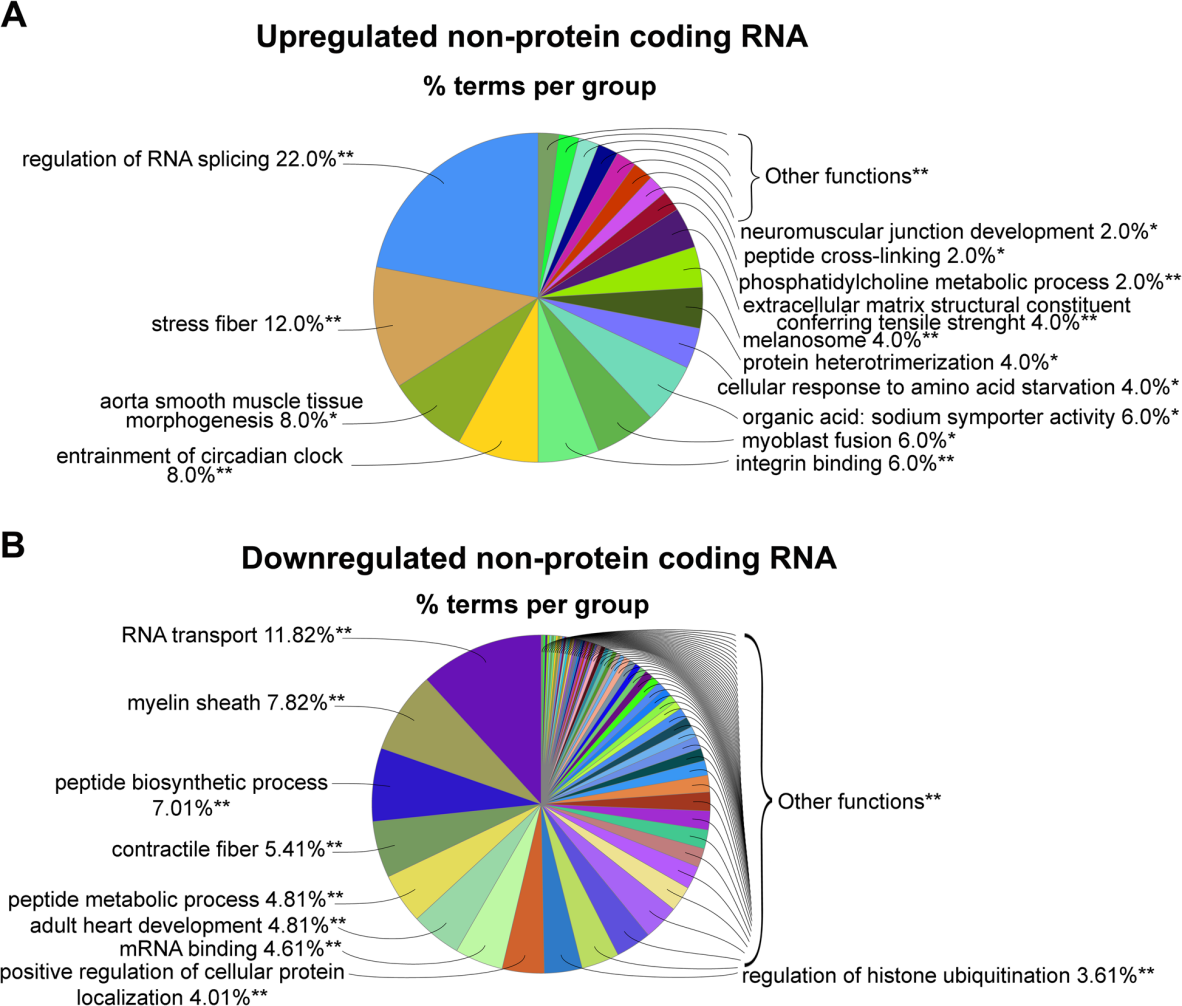
Functional analysis of the non-protein coding RNA differentially regulated by RGMa treatment. For this analysis, we considered upregulated DETs that do not encode proteins. Pie analysis of the GO enrichment, showing the most frequent terms, including cellular component, biological process, molecular function, and immune system process, and KEGG GO terms that were **A** upregulated and **B** downregulated. The right-sided hypergeometric test was used in statistical inference, and the Benjamini–Hochberg method was applied for a *p*-value correlation (*p* < 0.05). The analysis was conducted using the plugin ClueGO (v.2.5.4) for Cytoscape (v3.7.1)

The non-coding RNA found as DETs that were upregulated by RGMa treatment were mostly involved with the ‘regulation of RNA splicing,’ ‘stress fibre,’ ‘myoblast fusion,’ and ‘integrin binding’ (Fig. 3A), while ‘regulation of RNA transport’ and ‘peptide biosynthetic process’ were enriched among the downregulated non-coding DETs (Fig. 3B).

### GO pathway enrichment analysis of the protein coding RNA found as DETs

**Fig. 4:**
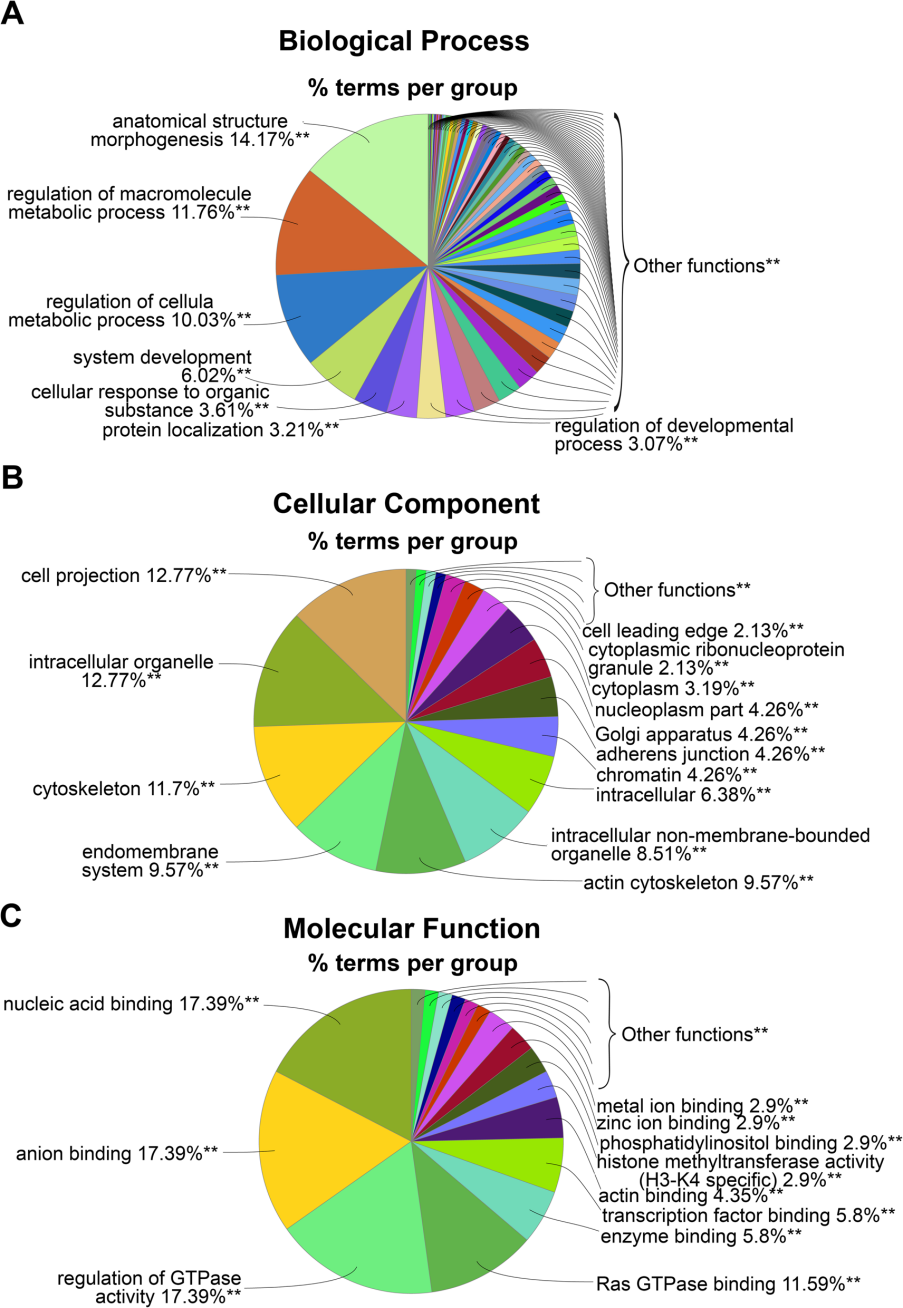
Functional analysis of the protein coding RNA upregulated by RGMa. For this analysis, we considered the DETs that encode proteins that were found to be upregulated (FC > 1) by the treatment with RGMa, compared to the control. **A-C** Pie chart analysis of the three GO categories used to classify the upregulated protein coding transcripts. The right-sided hypergeometric test was used in statistical inference, and the Benjamini–Hochberg method was applied for a *p*-value correlation (*p* < 0.0001). The analysis was conducted using the plugin ClueGO (v.2.5.4) for Cytoscape (v3.7.1)

DETs were characterized based on the Gene Ontology (GO) terms to identify the pathways that were enriched among the up- and downregulated transcripts. The enriched GO terms for the protein coding upregulated DETs were mostly related to the following biological processes: ‘morphogenesis,’ ‘metabolism,’ and ‘developmental regulation of muscle cell’ (Fig. 4A). Related to the cellular components, RGMa treatment could induce the upregulation of transcripts associated with ‘cytoskeleton,’ ‘cell projection,’ ‘endomembrane system,’ ‘adherens junction,’ ‘nucleus,’ and ‘nucleoplasm’ (Fig. 4B); and related to molecular function, transcripts were grouped as ‘nucleic acid binding,’ ‘transcription factor binding,’ and ‘regulation of GTPase’ and ‘Ras GTPase activity’ (Fig. 4C).

**Fig. 5:**
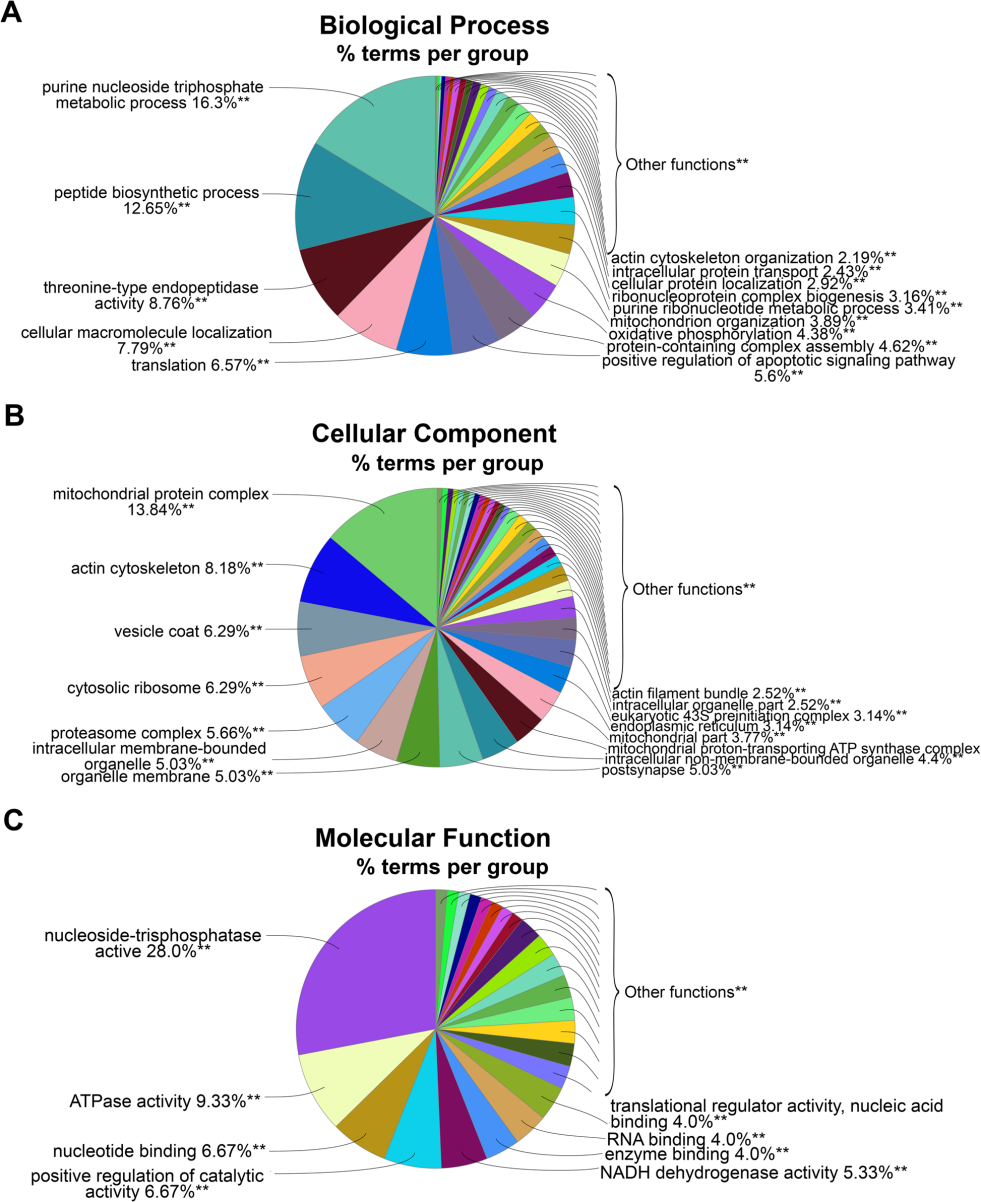
Functional analysis of the protein coding RNA downregulated by RGMa. For this analysis, we considered the DETs that encode proteins and were found to be downregulated (FC < 1) in the RGMa treated group, compared to the control one. **A-C** Pie chart analysis of the three GO categories for downregulated DETs. **D** Functionally grouped network of enriched categories for expressed transcripts, annotated for ‘biological process,’ ‘cellular component,’ and ‘molecular function’ GO terms. The right-sided hypergeometric test was used in statistical inference, and the Benjamini–Hochberg method was applied for a *p*-value correlation (*p* < 0.001). The analysis was conducted using the plugin ClueGO (v.2.5.4) for Cytoscape (v3.7.1)

A different pattern was found with the classification of the protein coding downregulated DETs; terms related to ‘metabolism’ and ‘tissue survival’ were the most downregulated after RGMa treatment. ‘Purine nucleoside triphosphate metabolic process,’ ‘peptide biosynthetic process,’ ‘translation,’ and ‘positive regulation of apoptotic signaling pathway’ were the most enriched terms of biological processes (Fig. 5A). Cellular components were mostly associated with ‘mitochondria protein complex,’ ‘actin cytoskeleton,’ ‘cytosolic ribosome,’ ‘proteasome complex,’ and ‘vesicle coat’ (Fig. 5B), while those found to be associated with molecular function were grouped in ‘activity of nucleoside-triphosphatase,’ ‘ATPase,’ and ‘positive regulation of catalysis’ categories (Fig. 5C).

### Cell adhesion and hypertrophy-associated terms

**Fig. 6:**
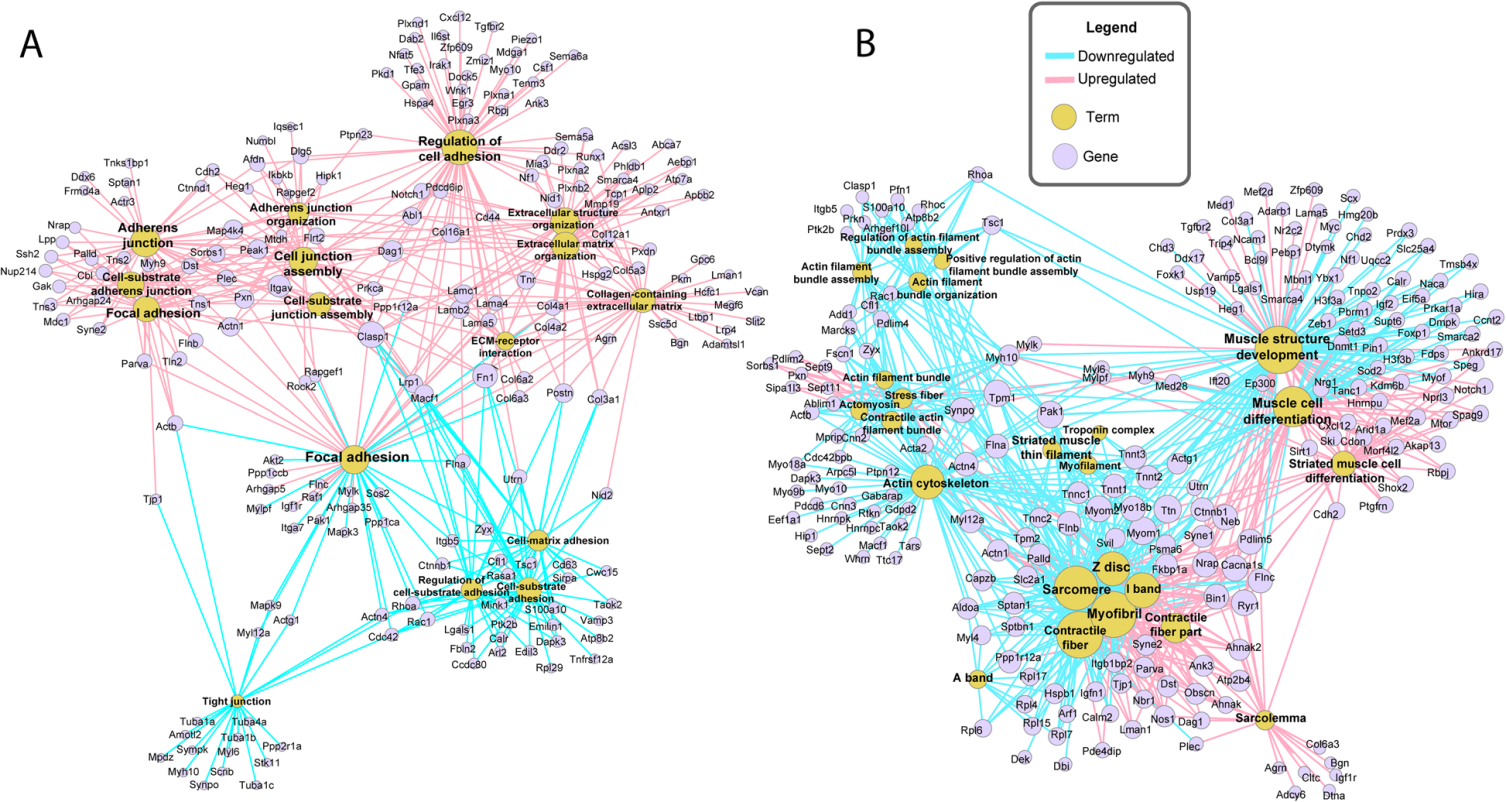
Muclei accretion and muscle-related enriched terms from the functional analysis of all DETs in response to RGMa. GO enrichment and the network analysis of DETs was performed using the software ClueGO. Terms were selected for network analysis related to nuclei accretion **A** and to muscle differentiation and structure **B**. The right-sided hypergeometric test was used in statistical inference, and the Benjamini–Hochberg method was applied for a *p*-value correlation (*p* < 0.001). The network was designed using the ForceAtlas2 algorithm and node size represents network centrality which was calculated using Eigenvector Centrality algorithm

We selected GO terms associated with “cell adhesion” and with “skeletal muscle structure” and “hypertrophy”, from both up and downregulated DETs, for a network analysis (Fig. 6).

Our analysis revealed a number of upregulated transcripts were associated with GO terms including ‘adherens junctions’, ‘cell-substrate adherens junction’, ‘adherens junction assembly’ and ‘adherens junction organization’, ‘cell-substrate junction assembly’, ‘regulation of cell adhesion’, ‘extracellular matrix organization’, ‘collagen-containing extracellular matrix’; while downregulated transcripts were mainly associated with ‘Focal adhesion’, ‘regulation of cell-substrate adhesion’, ‘cell-substrate adhesion’, ‘cell–matrix adhesion’ and ‘tight junction’ (Fig. 6A).

Related to muscle term, we could find upregulated transcripts associated with GO terms including ‘contractile fiber part’, ‘muscle cell differentiation’ and ‘striated muscle cell differentiation’; while the transcripts found as downregulated were mostly associated with the following GO terms: ‘sarcomere’, ‘Z disk’, ‘I band’, ‘myofibril’, ‘contractile fiber’, ‘myofilament’, ‘striated muscle thin filament’, ‘troponin complex’, ‘actin cytoskeleton’, ‘contractile actin filament bundle’, ‘stress fiber’, among others (Fig. 6B).

### RNA-seq validation

**Fig. 7:**
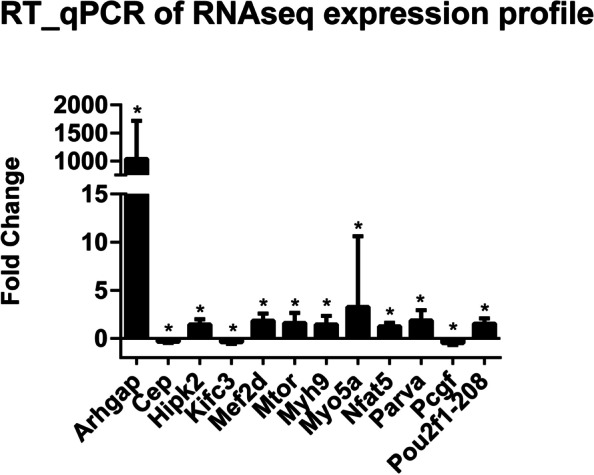
Validation of the RNA-seq expression profiles by qPCR. A subset of twelve DETs that were upregulated and downregulated by RGMa treatment during muscle differentiation were used to validate the obtained RNA-seq expression data. Transcripts were selected by their expression and their known association with muscle hyperplasic or hypertrophic phenotypes. Expression patterns indicate agreement between the two methods and *, significance of p-adj < 0.05

We chose 12 DET isoforms to validate our RNA-seq data and analysis by qPCR. *Arhgap35-202 and 201*, *Hipk2-206*, *Mef2d-202, 204 and 203*, *mTOR-202*, *Myh9-201*, *Myo5a-214*, *Nfat5-206, 208 and 214*, *Parva-203*, and *Pou2f1-208* were selected from the upregulated DETs isoforms, and *Cep164-170, Kifc3-202* and *Pcgf1-201* isoforms were chosen from the downregulated ones. The qPCR results showed a total concordance with the RNA-seq analysis (Fig. 7).

## Discussion

Although originally identified as a guidance clue for axonal growth, RGMa has been identified as playing roles in a number of different biological processes, including during myogenesis. *RGMa* transcripts could be found in chicken somites at the origin site of the muscle and satellite cell precursors [24]. In adult muscle, RGMa was found in regions of the sarcolemma and sarcoplasm, with an expression pattern similar to sarcomeric proteins [25]. Initial functional studies revealed that RGMa can induce myonuclear accretion and hypertrophy of myotubes, suggesting that this axon guidance molecule might be involved with the mechanisms that modulate skeletal muscle cell size [25].

However, the molecular mechanisms induced by RGMa during these important muscle phenotypes have not been clarified thus far. RGMa exerts its canonical effects through the type-I transmembrane neogenin receptor [6, 7, 9, 37], but it can also work as a bone morphogenetic protein (BMP) co-receptor, as it shares the same binding site in BMP-R1A with BMP ligands [15]. Notably, both signaling pathways seem to be active in skeletal muscle cells, inducing similar phenotypes in controlling the cell size, but these effects were never investigated in the context of having RGMa as a possible ligand. Using RGMa recombinant proteins in C2C12 cells, we could not clearly elucidate if RGMa effects were induced via neogenin and/or BMP signaling pathways, possibly because these receptors do not have RGMa as an exclusive ligand [38]. For this reason, in this work, the transcriptome of C2C12 cells was sequenced after being treated with RGMa recombinant protein during the late differentiation stage to detect the transcripts that had their expression modulated by this axon guidance molecule during the differentiation of skeletal muscle cells.

A database composed of 23,856 transcripts expressed during C2C12 differentiation was generated. Sequenced biological triplicates from treated and control groups were found to be homogeneous, conferring internal consistency and reproducibility of replicate samples. Three technical replicates are considered a sufficient for a reliable quantitative inferential analysis [39]. From these expressed transcripts, 2,195 were modulated by RGMa treatment, with 943 upregulated and 1,252 downregulated.

From this database, it was noted that RGMa was able to modulate the expression of five RNA biotypes. The most frequent RNA biotype modulated by RGMa was ‘protein coding,’ which included ORF containing transcripts. However, a significant portion of DETs were included in categories involved with the regulation of gene expression, including in the ‘nonsense mediated decay’ (composed of transcripts with a premature stop codon) and ‘processed transcript’ (composed of ‘retained intron RNA’, ‘antisense’ and ‘ncRNA’) biotypes. According to Wong et al. (2013), a number of transcripts must be destroyed to permit developmental transitions during differentiation [40]. Therefore, this data suggests that RGMa treatment induced the regulation of the genes that were being expressed during the differentiation stages using these particular molecular mechanisms, allowing the adaptation of these cells to reach terminal differentiation.

Additionally, our analysis also revealed that RGMa could differentially induce the expression of alternatively spliced transcripts. *Pou2F1* (Pou Class 2 Homeobox 1, also known as Oct-1) isoforms were found to be the most upregulated DET (Pou2f1-208, ENSMUST00000160260.9), as well as the most downregulated DET (Pou2f1-205, ENSMUST00000111427.9) by RGMa treatment in skeletal muscle cells. Although the specific functions of each of these isoforms have not been described thus far, it is known that Pou2F1 is an ubiquitously expressed member of the Pou transcription factor family and is associated with a plethora of processes, including the activation of some snRNA, histone H2B, immunoglobulins, and other housekeeping genes [41], the regulation of the circadian clock [42], and glycolytic metabolism [43]. In skeletal muscle cells, this transcription factor was associated with the activation of pro-inflammatory immune response in patients with myalgia [44] and with MyHC IIB expression, when associated with MEF2 and the serum response factor (SRF) [45–47]. Pou2F1 was also identified on a slow skeletal muscle troponin I promoter in Gaoyou duck skeletal muscle [48]. In addition to *Pou2F1*, multiple isoforms for *myoferlin* (*Myof*), *myosin heavy chain 10* (*Myh10*), *myosin IXB* (*Myo9b*), *titin* (*Ttn*), *tensin 2* (*Tns2*), *supervillain* (*Svil*), and *chromodomain helicase DNA binding protein 2* (*Chd2*) were also found as modulated by the RGMa treatment in C2C12 cells; these genes are of wide importance for development, differentiation, and maintenance of skeletal muscle cells.

### RGMa treatment modulated the expression of muscle hypertrophic markers

The protein coding DETs were analyzed to determine how RGMa induces hypertrophic and nuclear accretion effects on skeletal muscle cells.

Our transcriptome database showed that RGMa induced the expression of the mammalian target of rapamycin (*mTOR*) transcript, which is a common factor from different pathways that culminate with skeletal muscle hypertrophy [27, 49–53]. RGMa could specifically induce *mTOR* transcript isoform 202 (ENSMUST00000103221.10), suggesting a new mechanism for this isoform in these cells. The effect of RGMa on *mTOR* expression was also confirmed by qPCR. mTOR exerts its effects as part of two complexes, termed mTORC1 and mTORC2. Increased mTORC1 activity can positively regulate muscle protein synthesis via S6K1 and also inhibit its negative regulation when working via 4EBP1 [27, 54]. TSC1, in a complex with TSC2, is responsible for the negative regulation of mTORC1 signaling, inhibiting the nutrient-mediated or growth factor-stimulated phosphorylation of S6K1 and 4EBP1 [53]. Furthermore, *TSC1-204* (ENSMUST00000113870.3) was also highly downregulated by RGMa treatment in skeletal muscle cells. The inhibition of TSC1/2 protein synthesis resulted in rapid activation of mTORC1 signaling independent of Akt [53, 55]. The hypertrophic effects observed by RGMa treatment could then be a result of the inhibition of the *TSC1* transcript and of the induction of *mTOR* expression, which are both crucial for muscle growth. Additionally, although the TSC1/2 complex is not physically associated with mTORC1, it is required for mTORC2 activation and consequently, for Akt phosphorylation, in a manner that is independent of its GTPase-activating protein activity toward Rheb [56]. Thus, the inhibition of TSC1 by RGMa suggests that RGMa simultaneously works to prevent mTORC2 activation. The fact that TSC1 inhibition contributes to mTORC1 activation independently of Akt, as well as to mTORC2 inhibition, resulting in the loss of Akt stimulation [55], might explain why Akt was not induced by RGMa in skeletal muscle cells. Our results suggest that *mTOR* upregulation in response to RGMa is independent of Akt phosphorylation.

Other factors associated with the mTORC pathway were also dysregulated by the RGMa treatment and could contribute to including this axon guidance molecule in an alternative muscle hypertrophic pathway. For example, RGMa could induce the expression of the *phospholipase D1* (*Pld1-202,* ENSMUST00000120834.8) transcript, which was found to be an activator of mTORC1 [50, 57].

RGMa could also induce the upregulation of members of the Myocyte Enhancer Factor 2 (Mef2) family, specifically *Mef2a-204* (ENSMUSG00000030557.17) and *Mef2d-204* (ENSMUSG00000001419.17) isoforms. Mef2 transcription factors activate many muscle-specific growth factor-induced genes and regulate muscle cell differentiation and muscle embryonic development [58–60]. Mef2 can also act as a nodal point for remodeling programs in metabolic gene expression, fiber-type switching, and skeletal muscle regeneration [58, 59, 61]. *Mef2a* upregulation can also contribute to terminal differentiation and myoblast fusion, which is also consistent with the present GO term analysis and with the RGMa muscle phenotype [25, 38]. *Mef2a*, *Mef2c,* and *Mef2d* deleted in combination in satellite cells abolished skeletal muscle regeneration after cardiotoxin injury [59].

Our RNA-seq database suggested other hypertrophic mechanisms that could be regulated by RGMa treatment, including the upregulation of *Sirtuin 1* (*Sirt1*), which is known to regulate protein degradation via FoxO inhibition [62]; the upregulation of *Nos1,* which interacts with Sirt1 [63]; or the downregulation of genes that promote muscle protein degradation, such as the *activating transcription factor 4* (*Atf4)* [64, 65].

### RGMa treatment also modulated the expression of genes associated with nuclei accretion

We have also searched for genes associated with myonuclear accretion that were modulated by RGMa treatment in C2C12 cells. Among these, *cadherin2* (*Cdh2, ENSMUST00000025166.13*), *integrin alpha-V* (*Itgav, ENSMUST00000141725.2*), *neural cell adhesion molecule* (*NCAM, ENSMUST00000166811.8*), *calcium voltage-gated channel subunit alpha1S* (*Cacna1s, ENSMUST00000112068.9*), *actinin alpha 1* (*Actn1, ENSMUST00000167327.1*), *disabled homolog 2* (*Dab2, ENSMUST00000080880.11*), *myoferlin* (*Myof, ENSMUST00000224560.1, ENSMUST00000041475.15,* ENSMUST00000224518.1*),* the myosins *Myo5a (ENSMUST00000155282.8, ENSMUST00000123128.7)*, *Myo10 (ENSMUST00000022882.11*, ENSMUST00000110457.7, ENSMUST00000125667.2), *Myh9* (ENSMUST00000016771.12), Myh*10 (ENSMUST00000102611.9) phosphatase,* and *actin regulator 4* (*Phactr4, ENSMUST00000136711.1*) were upregulated by RGMa treatment.

*Myof*, for example, is a member of the Ferlin protein family, highly expressed in myoblasts during the pre-fusion phase of differentiation and in myofibers, especially during regeneration after injury [66–68]. It is associated with fusion events and intracellular trafficking in muscle, including myoblast fusion, vesicle traffic, membrane repair, and endocytic recycling [69].

*Myh9* and *Myh10* are equally fundamental for the positive regulation of cell–cell adhesion and myoblast fusion [70]. *Myh9* is known as non-muscle myosin heavy chain IIa (NMMHC-IIA), while Myh10 is the non-muscle myosin heavy chain IIb [71]. These myosins are expressed in most cell types, working as motor proteins in a variety of processes requiring contractile force, such as cytokinesis, cell migration, polarisation and adhesion, maintenance of cell shape, and signal transduction [72–74]. In skeletal muscle cells, non-muscle myosins drive myoblasts to align and fuse to form multinucleated myotubes [70, 75]. The knockdown of these myosins inhibit the change of the myoblast shape, interfering with cell–cell adhesion and fusion [70].

Dab2 plays an important role as a modulator of cell–cell interactions, as it is a clathrin adaptor and can mediate integrin signaling [76]. In the musculature, *Dab2* was detected during early myogenic differentiation [77, 78]. Shang et al. (2020) showed that *Dab2* expression is upregulated in C2C12 myoblast during the differentiation in myotubes, and its knockdown resulted in reduced myoblast fusion and fewer myotubes. Besides, Dab2 overexpression could enhance the myotube formation and also restore the myotube differentiation capacity of its knockdown [79].

The *calcium voltage-gated channel subunit alpha1 S* (*Cacna1s-202,* ENSMUST00000112068.10) encodes one of the five subunits of the L-type voltage-dependent calcium channel in skeletal muscle cells. In the musculature, calcium is generally related to muscle contraction and muscle relaxation [80–82]. However, the regulation of calcium influx into muscle cells plays a critical role in muscle differentiation [82, 83]. Intracellular calcium is able to regulate transcription factors necessary for myotube fusion [83, 84], while its reduction inhibits myoblast differentiation [85]. The upregulation of *Cacna1s* in response to RGMa treatment suggests an association with the regulation of intracellular calcium, which is important for the myoblast fusion process and myotube contraction.

## Conclusion

The current work allowed us to unravel some molecular mechanisms that were altered in skeletal muscle cells after treatment with RGMa, especially those associated with muscle nuclei accretion and hypertrophy. Our analysis suggested that RGMa induced cell hypertrophy via (i) upregulation of hypertrophic markers, (ii) downregulation of inhibitors of hypertrophic pathways, (iii) downregulation of transcripts related to the positive regulation of muscle atrophy, and (iv) upregulation of transcripts that negatively regulate atrophy. At the same time, transcripts associated with known myoblast fusion pathways were also found to be modulated by RGMa, mainly those related to cell–cell adhesion pathways.

Our results provide comprehensive knowledge of skeletal muscle transcriptional changes and pathways in response to RGMa treatment.

## Material and methods

### Cell culture and differentiation

The lineage of immortalized mouse myoblasts C2C12 (ATCC® CRL1772™) was cultured at 37 °C and 5% CO_2_ in growth medium (GM), composed of DMEM (Dulbecco’s Modified Eagle’s Medium) with high glucose and L-glutamine (Gibco), supplemented with 10% fetal bovine serum (FBS, Gibco) and 1% penicillin, streptomycin, and amphotericin B solution (Gibco). Myogenic differentiation was induced in differentiation medium (DM), composed of DMEM, supplemented with 2% horse serum (Gibco) and 1% penicillin, streptomycin, and amphotericin B. For growth or differentiation conditions, the medium was replaced every 2 days.

### RGMa recombinant protein treatment

C2C12 cells were seeded at 2 × 10^4^ cells per well in 24-well plates and cultivated in GM at 37 °C and 5% CO_2_. After reaching 90–100% confluency, cells were induced to differentiate in DM for 72 h (Fig. 1A). DM was then replaced with fasting medium (FM), composed of DMEM supplemented with 0.2% FBS, and cells were incubated at the same conditions for 3 h. Subsequently, C2C12 were treated with 50 ng/ml mouse RGMa recombinant protein (R&D Systems) in FM and incubated for an additional 48 h, as previously described [38]. The recombinant protein was omitted in the control samples.

### Total RNA isolation and cDNA synthesis

Cells were harvested in TriReagent (Sigma Aldrich) as pools of three wells in triplicate. Total RNA isolation was performed according to the manufacturer's instructions. Sample integrity, purity, and concentration were evaluated by electrophoresis in 1% agarose gel and in NanoDrop® ND-1000 UV/Vis Spectrophotometer, respectively.

The quality of the total RNA was also evaluated in a Bioanalyzer (Agilent) before being submitted to sequencing. Values for RNA integrity number (RIN) ranging from 8 to 10 were considered suitable for RNA-seq.

### RNA-seq library preparation and next-generation sequencing (NGS)

For cDNA library construction, 2 μg of total RNA were treated with 1U of DNaseI amplification grade (Invitrogen) and purified according to the TruSeq Stranded mRNA Sample Prep LS Protocol of Illumina (http://grcf.jhmi.edu/hts/protocols/mRNA-Seq_SamplePrep_1004898_D.pdf), using magnetic microspheres for messenger RNA separation. The purified mRNA was fragmented in Illumina buffer. Superscript III (Invitrogen) and oligo(dT) were used for reverse transcription of the first cDNA strand. The second strand was synthesized using the enzymes RNase H and DNA Polymerase I (Illumina). Molecule ends were treated with T4 DNA Polymerase and Klenow DNA Polymerase (Illumina), making them blunt. The 3’ end of the synthetized cDNA was phosphorylated with T4 PNK (Illumina) and adenylated with Klenow exo (Illumina). Adaptors were bound to cDNA ends, and the samples were purified and selected by size of 200 bp ± 25 bp after fractioning in agarose gel electrophoresis (QIAquick Gel Extraction Kit, QIAGEN). Purified cDNA was quantified by RT-qPCR using adaptor-specific oligonucleotides (Illumina).

Sequencing was performed using HiScanSQ (Illumina),—according to the manufacturer’s recommendations, and using the paired-end reads protocol. Each sample was sequenced until it reached around 34 million reads/library.

### Mapping RNA-seq data

Transcript quantification analysis was performed based on Salmon (version 0.13.1), an open-source and freely-licensed software (available at https://github.com/COMBINE-lab/Salmon [86]). Raw reads were used as an input to quantify transcripts in mapping-based mode. The current version of the mouse transcriptome (available at https://www.gencodegenes.org/mouse/release_M20.html) was used as a reference, which includes all RNA categories used to classify the transcripts obtained in this work.

### Statistical RNA-seq

Statistical analysis was performed using the DESeq2 package of R Bioconductor [87]. An adjusted *p*-value with a false discovery rate (FDR) correction (Benjamini and Hochberg, 1995) of 5% was calculated and used to control false-positive significance in transcript expression variation. Log2(fold change) > 0 and log2(fold change) < 0 were selected as the threshold to show an increase or decrease in transcript expression of treated groups relative to the control group.

### Transcript expression pattern and RNA-seq quality analysis

Transcript-specific normalisation was performed to remove disparities in the base means correlations and to eliminate the noise of transcripts with low expression.

Normalised transcripts were plotted in MA form using the DESeq2 package to generate a scatter plot of log2 fold changes < 0 and > 0 versus the mean of normalised counts of transcripts, considering DE those with FDR < 0.05. The correlation of each sample and the clustering of the treated and control groups was performed by calculating the PCC of normalised read-counts.

### Functional RNA-seq analysis

The GO enrichment and the network analysis of DETs was performed using the software ClueGO v.2.5.4 [88] and Gephi v 0.9.2 (https://ojs.aaai.org/index.php/ICWSM/article/view/13937). The right-sided hypergeometric test was used to identify overrepresented GO terms and the BenjaminiHochberg method was used for the correction of the *p*-values (*p* < 0.001). The Ensembl Transcript ID of the DETs was used as input for ClueGO analysis. Terms were selected for network analysis by related to nuclei accretion (Fig. 6A) and related to muscle differentiation and structure (Fig. 6B). The network was designed using the ForceAtlas2 algorithm and node size represents network centrality which was calculated using Eigenvector Centrality algorithm.

The heatmap graph was obtained using the D3Heatmap package (https://www.rdocumentation.org/packages/d3heatmap/versions/0.6.1.2), using ID ensemble transcripts as an input (of clue go output for muscle associated terms) and the correlated base mean expression.

### Primer design and qPCR

qPCR was performed for the specific upregulated isoforms of *Arhgap35-202 and 201*, *Hipk2-206*, *Mef2d-202, 204 and 203*, *mTOR-202*, *Myh9-201*, *Myo5a-214*, *Nfat5-206, 208 and 214*, *Parva-203*, *Pou2f1-208* and for the downregulated isoforms of *Cep164-170, Kifc3-202* and *Pcgf1-201*, that were selected due to their importance for muscle phenotypes, as well as by their relevance between the more enriched terms.

The multiline interface (http://multalin.toulouse.inra.fr/multalin/) was used for the alignment of genes with some specific isoforms up and others downregulated by RGMa. The non-consensus sequences among them were selected to avoid undesired isoforms and the consensus ones were used to obtain an amplicon of up to 250 bp for the chosen isoforms. Primer 3.0 software was used for primer design. Manual primers were designed for small specific strings.

cDNA was synthesized using 1 μg of total RNA following the recommendations of the RevertAid™ H Minus First Strand cDNA Synthesis kit (Fermentas).

qPCR was performed in the Rotor-Gene RT-qPCR system (Qiagen), using the iTaq Universal Sybr Green Supermix (Bio Rad) and 0.4–0.8 μM of each primer for a final volume of 10 μl. GAPDH was used as a housekeeping gene. The analysis of differential gene expression was performed using REST 2009 (Relative Expression Software Tool, V.2.0.13) software via randomisation tests (Pair Wise Fixed Reallocation Randomisation Test) [89] with 95% significance.

## Supplementary Information

**Additional file1:** **Supplementary file 1.** Differentially expressed transcripts modulated by RGMa treatment during the differentiation of C2C12 cells.

**Additional file 2:** **Supplementary file 2.** Differentially expressed genes modulated by RGMa treatment during the differentiation of C2C12 cell.

## Data Availability

The raw transcriptome sequencing data (RNA-seq) from the technical replicates of C2C12 myoblast treated with RGMa are available under the NCBI-BioProject submission code PRJNA730936. The datasets supporting the conclusions of this article are included within the article and its additional files.

## Abbreviations

4EBP1: Ukaryotic Translation Initiation Factor 4E Binding Protein 1
Acap: ArfGAP With Coiled-Coil, Ankyrin Repeat And PH Domains 1
Actn1: *Actinin* *alpha 1*
Akt: Serine/Threonine Kinase 1
ALK5: Activin A Receptor Type II-Like Protein Kinase Of 53kD, also Known as Transforming Growth Factor Beta Receptor 1
Ank3: Ankyrin 3
Ap2a1: Adaptor Related Protein Complex 2 Subunit Alpha 1
Arhgap-35: Rho GTPase Activating Protein 35
Atf4: *Activating transcription factor 4*
BMP: Bone Morphogenetic protein
BMP-R1A: BMP type I receptor A
C2C12: Immortalized Mouse Myoblast cell line
Cacna1s: Calcium voltage-gated channel subunit alpha1S
Cep164: Centrosomal Protein 164
Cdh2: Cadherin2
cDNA: Complementary DNA
Chd2: *Chromodomain* *helicase DNA binding protein 2*
C-RGMa: RGMa C-terminal domain
Dab2: Disabled homolog 2
DE: Differentially expressed
DET: Differentially expressed transcripts
DM: Differentiation medium
DMEM: Dulbecco’s Modified Eagle’s Medium
FBS: Fetal bovine serum
FDR: False Discovery Rate
FM: Fasting medium
FoxO: Forkhead Box Protein O
GDPH: Glyceraldehyde 3-phosphate dehydrogenase
GM: Growth medium
GO: Gene Ontology
GPI-anchor: Glycosylphosphatidylinositol-anchor
GTP: Nucleotide guanosine triphosphate
Hipk2: Homeodomain Interacting Protein Kinase 2
Iqsec1: IQ Motif And Sec7 Domain ArfGEF 1
Itgav: Integrin alpha-V
KEGG: Kyoto Encyclopedia of Genes and Genomes
Kifc3: Kinesin Family Member C3
lncRNA: Long non coding RNA
Mau2: MAU2 Sister Chromatid Cohesion Factor
Mef2: Myocyte Enhancer Factor 2
mRNA: Messenger RNA
mTOR: Mechanistic Target Of Rapamycin Kinase
mTORC1/2: Mechanistic target of rapamycin complex 1/2
Myh9: *Myosin heavy chain 9*
Myh10: Myosin heavy chain 10
Myof: Myoferlin
Myhc IIB: Myosin heavy chain IIb
Myo5a: Myosin VA
NCAM: Neural cell adhesion molecule
NCBI: National Center for Biotechnology Information
ncRNA: Non coding RNA
NGS: Next Generation Sequencing
NMD: Nonsense Mediated Decay
Nos1: Nitric Oxide Synthase 1
Nrap: Nebulin Related Anchoring Protein
N-RGMa: RGMa N-terminal domain
Oct-1: Octamer-Binding Transcription Factor 1
Oligo(dT): Oligonucleotide (deoxythymine)
ORF: Open reading frame
Parva: Parvin Alpha
PCC: Pearson correlation coefficient
Pcgf1: Polycomb Group Ring Finger 1
Phactr4: Phosphatase and actin regulator 4
Picalm: Phosphatidylinositol Binding Clathrin Assembly Protein
Pld1: Phospholipase D1
Pou2F1: Octamer-Binding Transcription Factor-1, also known as Oct1
Rab1a: RAB1A, Member RAS Oncogene Family
Rac: Family Small GTPase 1
RGD: Tripeptide Arg-Gly-Asp
RGMa: Repulsive Guidance Molecule a
Rho: Rhodopsin
RIN: RNA integrity number
RNA: Ribonucleic acid
RNA-seq: RNA sequencing
RT-qPCR: Quantitative reverse transcription polymerase chain reaction
S6k1: Ribosomal Protein S6 Kinase B1
Scaper: S-Phase Cyclin A Associated Protein In The ER, also known as Zinc Finger Protein 291
Sirt1: Sirtuin
Smad2/3: Mothers Against Decapentaplegic Homolog 2/3
snRNA: Small nuclear RNA
SRF: Serum response factor
Svil: Supervillain
T4 PNK: T4 Polynucleotide Kinase
Tns2: Tensin2
Ttn: Titin
Tbc1d25: TBC1 Domain Family Member 25
TEC: To be Experimentally Confirmed
TGF β1: Transforming Growth Factor β1
Tnpo3: Transportin 3
Tsc1: Tuberous Sclerosis 1 Protein
Tsc2: Tuberin
